# Novel Insights into Metagenomic-Assembled Genomes from Layer Chicken Housing Environment

**DOI:** 10.3390/ijms27177732

**Published:** 2026-08-28

**Authors:** Awais Ghaffar, Mohamed Faizal Abdul-Careem

**Affiliations:** 1 Faculty of Veterinary Medicine, University of Calgary, 3280 Hospital Dr NW, Calgary, AB T2N 4Z6, Canada; 2 Khan Bahadar Choudhry Mushtaq Ahmed, College of Veterinary and Animal Sciences, Narowal, University of Veterinary and Animal Sciences, Lahore 51600, Pakistan

**Keywords:** metagenomic-assembled genome, layer chicken, unclassified bacterial species, poultry barn air, poultry manure

## Abstract

Culture-independent techniques are playing a major role in exploring unique and novel microbial communities from complex ecosystems, leading to an outstanding impact on our basic understanding of the tree of life. Microbial communities are not extensively studied in layer chicken housing environments, particularly from the point of view of taxa carrying antimicrobial resistance genes, virulence genes and their functional potential. This study aimed to extract metagenomic-assembled genomes (MAGs) from the Illumina short-reads shotgun metagenomics sequenced data that originated from an Alberta poultry barn environment and then to study host tracking of antimicrobial resistance genes (ARGs) and the roles of genes involved in functions related to ammonia production, short-chain fatty acid (SCFA)-related pathways, sulfur metabolism, methane emission, stress and disinfectant-related pathways. A total of 251 high-quality MAGs were extracted, including 249 bacterial and two archaeal genomes from sequencing data of 30 metagenomic sequencing samples comprising 15 air and 15 manure samples collected from 15-layer farms. Interestingly 22 bacterial MAGs were not classified to species levels using GTDB-based classification. ARGs were mainly harbored by the genera *Staphylococcus*, *Alistepes*, *Romboutsia*, and *Enterococcus*. Bacteroides is a main taxon carrying ARGs in air samples. Ammonia production-related genes were mainly tracked in *Staphylococcus*, *Ruminococcus* and *Corynebacterium* genera. The assimilatory sulfate reduction genes responsible for sulfur metabolism and hydrogenase-related genes responsible for hydrogen cycling were traced from *Staphylococcus* originated from both air and manure. The current study provides characterizations of MAGs from a poultry housing environment by linking microbial taxa with virulence, resistance, and metabolic functions. The findings emphasize the role of microbiota in shaping gas emissions and AMR, with implications for poultry health and worker’s safety and the ultimate aim of sustainable poultry production.

## 1. Introduction

Poultry housing environments can shape the distribution of microbiota within a barn and the vicinity of a barn [1]. As only a few microbial communities are culturable from complex samples, metagenomic sequencing has provided a major change in how we reveal the diversity and functions of microbial communities from these specific ecosystems [2]. Culture-independent approaches have revolutionized microbiome research through the characterization of uncultivable microorganisms that inhabit complex ecosystems [3]. Shotgun metagenomics have proven to be a powerful technique that can be used to sequence the entire DNA from complex samples to reveal the overall depiction of DNA without prior preprocessing [4]. The use of this technique has advanced the understanding of the composition of complex microbial communities in various ecosystems [5].

Short reads from Illumina sequencing can be assembled into contigs which are grouped by binning tools into metagenomic-assembled genomes (MAGs) [6]. Following this approach, these MAGs can be further taxonomically classified and annotated into functional groups by curated databases, which can not only help us understand the ecology and role of microbial communities in an ecosystem but also the communities carrying potential antimicrobial resistance genes (ARGs) [7]. This knowledge and outcomes can be used to identify the role of potential taxa in the transmission of antimicrobial resistance (AMR), mapping the transmission potential and guiding interventions [8]. Furthermore, these studies have highlighted the potential of microbiota for their role in nutrient cycling, potential zoonotic risk, and environmental emissions from poultry [9,10].

Mobile genetic elements (MGEs) consist of dynamic components of the bacterial genomes that can play an important role in shaping adaptability and genetic diversity across various microbial populations [11]. Various MGEs, including transposons, plasmids, and prophages, have a role in facilitating genomic rearrangements and horizontal gene transfer [12]. By mobilizing functional genes across many bacterial species and environments, they act as commanding agents of ecological adaptation and evolution [11].

Although there are some reports, particularly from broiler chicken environments [13], only a small number of studies have used this method so far to recover archaeal and bacterial MAGs from the gut or housing environment of layer chickens from North America. Microbial communities are not extensively studied in layer chicken housing environments, particularly taxa carrying ARGs and the mobilome within Canada; however, such data is available from other countries [14]. Studying these communities can expand our knowledge and understanding of shared and unique taxa from layer chicken housing environments. Since chicken housing environments carry diverse microbial communities carrying various ARG subtypes and MGEs [15,16], through air and by spreading litter on agriculture soils, the microbial communities carrying these ARG subtypes can be dispersed to the outside environment and can be a serious one health concern [17]. The current study was aimed at extracting MAGs from DNA sequencing data that originated from the manure and air of layer chicken barns to find out the potential hosts of ARGs, for mobilome analysis, and to better understand the functional role of unique microbiota from chicken housing environments.

## 2. Results

A total of 2476 bins were recovered using metabat2 binning (Supplementary excel sheet_CheckM). Over one thousand (1012) bins recovered using Check M were of medium quality (50% completeness and had 10% contamination), while 251 bins recovered by Check M were of high quality (90% completeness and had 5% contamination). Two out of 251 bins were archaea, both belonging to *Methanobrevibacter woesei*, while the other 249 bins were of bacterial origin (Supplementary excel sheet_GTDB-tk classification). The recovered genomes of bacteria belonged to 11 phyla, 22 classes, 34 orders, 64 genera and 112 species. Interestingly 22/249 bins were not assigned to species levels (Table 1). Among 251 total MAGs, 109 originated from air samples and 142 originated from litter samples.

### 2.1. Network Analysis of ARGs and Taxa

**Figure 1:**
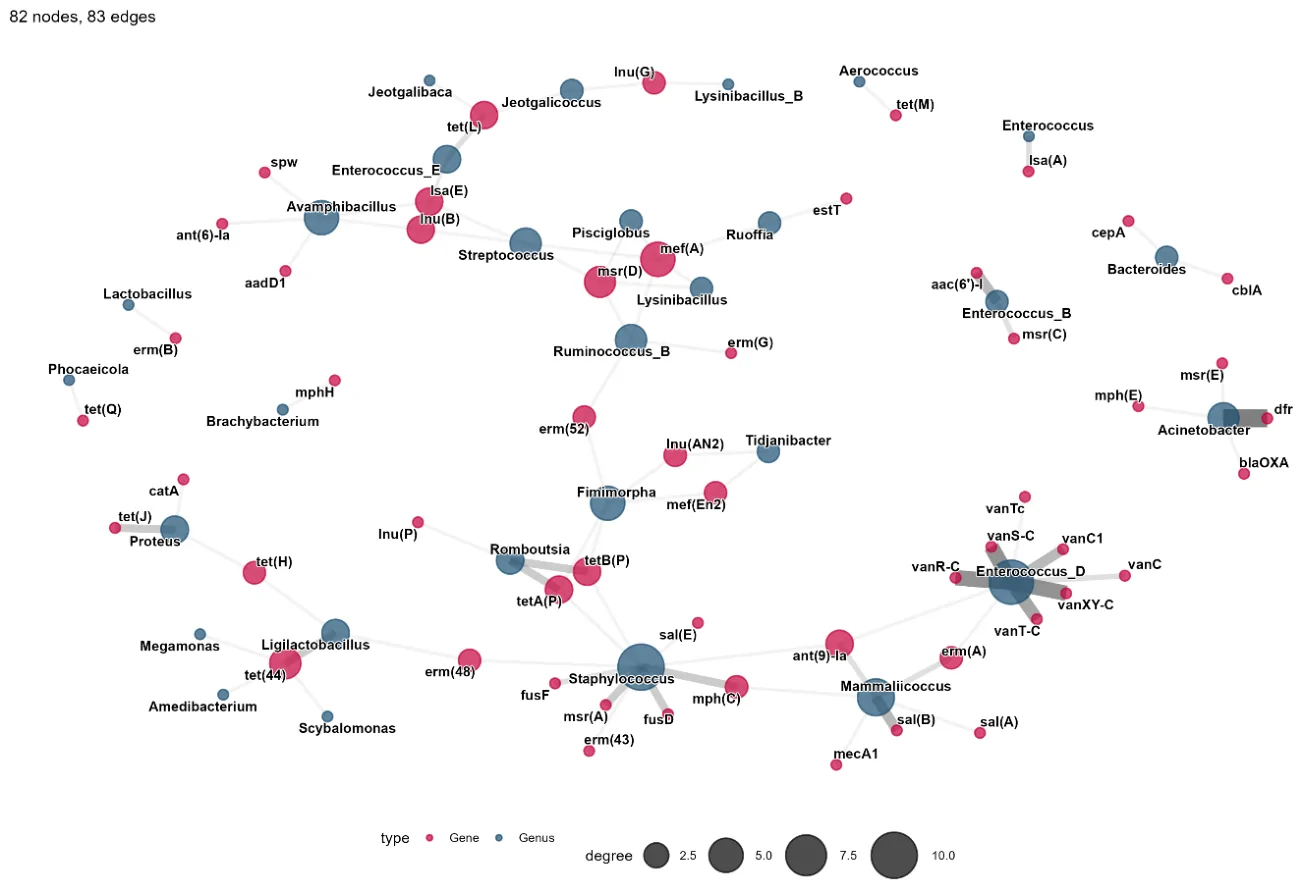
Bipartite ARG–genus association network from manure. Bacterial genera and ARGs are represented as two node types. Edges indicate observed detection of an ARG in MAGs assigned to the corresponding genus, and edge weight represents the number of detections supporting each association. No prevalence filter was applied. The network is a descriptive representation of observed ARG–genus associations; no correlations, P values, or inferential co-occurrence statistics were calculated.

**Figure 2:**
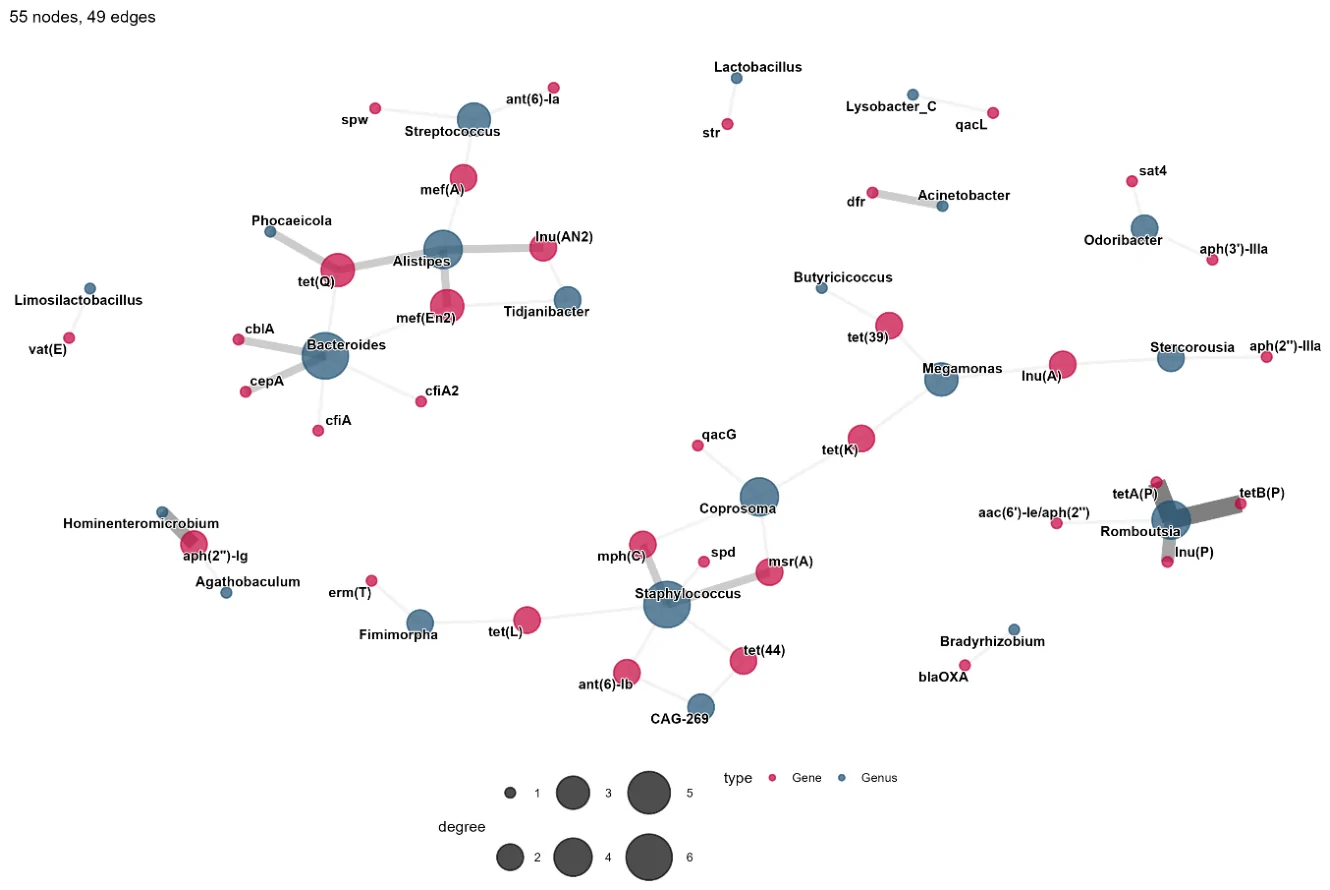
Bipartite ARG–genus association network from air. Bacterial genera and ARGs are represented as two node types. Edges indicate observed detection of an ARG in MAGs assigned to the corresponding genus, and edge weight represents the number of detections supporting each association. No prevalence filter was applied. The network is a descriptive representation of observed ARG–genus associations; no correlations, P values, or inferential co-occurrence statistics were calculated.

A network analysis was carried out to show the relatedness of taxa carrying ARG subtypes. Some interesting patterns were observed in the network analyses. The genera *Staphylococcus* and *Enterococcus* had most of the ARGs. It is important to note that these two genera were the major hosts carrying ARGs in litter (Figure 1), while Bacteroides along with *Staphylococcus* were the main taxa carrying ARGs in air samples (Figure 2). Some of the ARG subtypes were shared between more than one taxa. Some of the gene patterns were similar in both air and litter. In particular, *Staphylococcus* from both litter and air carried *msrA* and *mphC* gene subtypes linked to erythromycin resistance. Meanwhile all *van* gene clusters were only found from litter samples, which means some of the genes were only found in litter from the *Enterococcus* genus (Figure 1).

### 2.2. Taxonomy of MAGs

**Figure 3:**
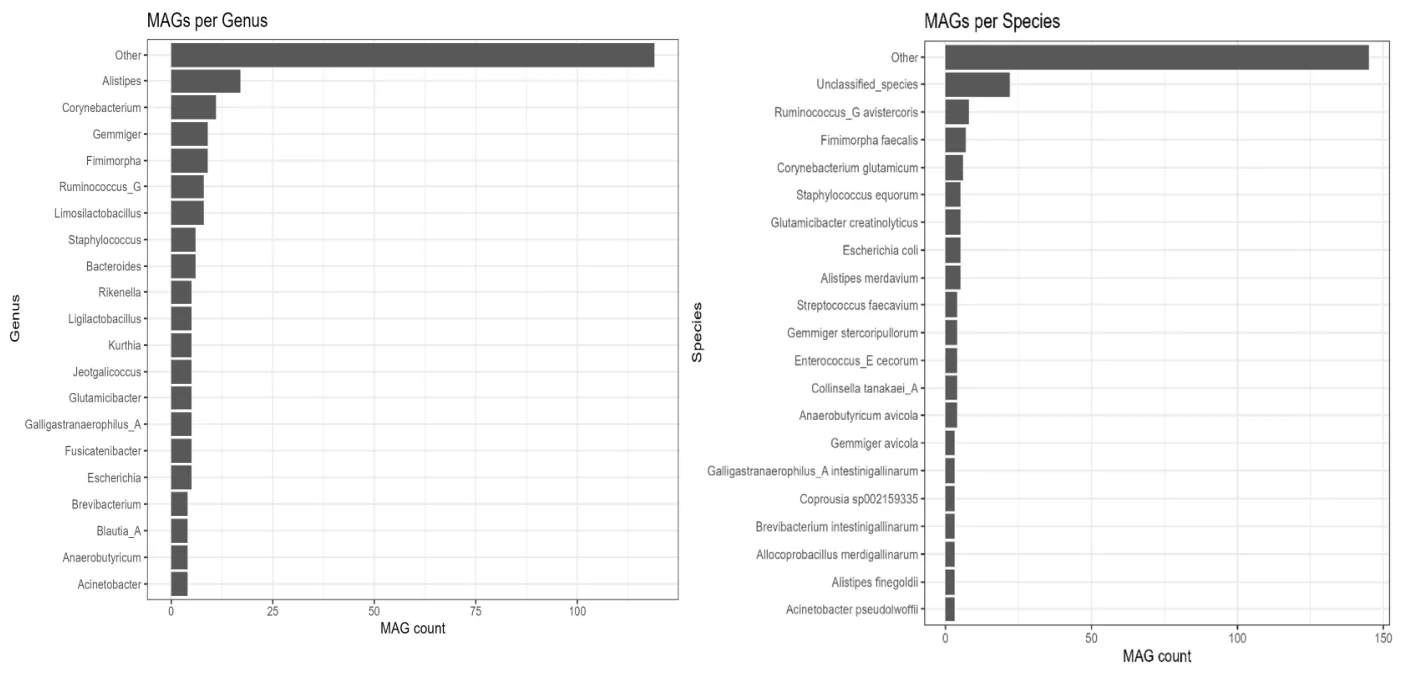
Top 20 genera and species of high-quality MAGs.

**Figure 4:**
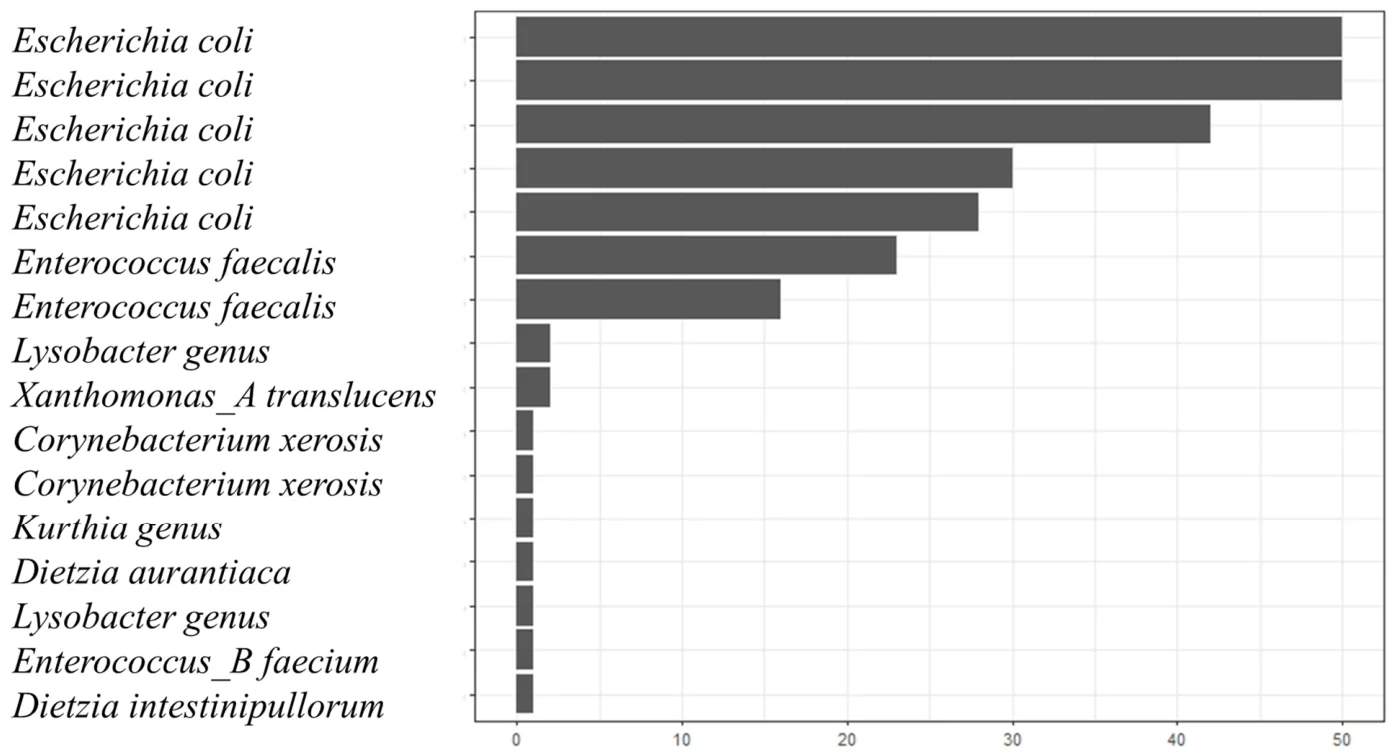
MAGs with virulence genes.

Out of 249 high-quality MAGs, 227 were assigned to the respective species levels using the GTDB-tk database, and interestingly among the 22 unclassified MAGs, 21 could be assigned to the genus level but could not be classified to a known species, whereas one MAG could not be assigned even at the genus level (Table 1). Overall, these genomes belonged to 11 phyla and 112 unique species. The 22 unclassified MAGs include five *Rikenella*, two *Sphingobacterium*, two *Coprenecus*, two *Lysobacter*, and one from *Sporosarcina*, *Tissierella*, *Cryptobacteroides*, *Corynebacterium*, *Lysinibacillus*, *Tissierella*, *Kurthia*, *Avamphibacillus*, *Neofamilia*, and *Yaniella*, with one MAG belonging to the family *Carnobacteriaceae* as an unclassified taxon (Figure 3). *Ruminococcus aveistercoris*, *Fimimorpha faecalis*, and *Corynebacterium glutamicum* were among the top three most found species from MAGs. Some of the MAGs carried virulence genes as well, and among them there were five *Escherichia coli*, three *Enterococcus faecalis*, and two *Corynebacterium xerosis*. *E. coli* harbored 50 virulence genes (Figure 4).

A total of 50 virulence-associated genes were detected across all MAGs, spanning multiple functional categories including adhesion and colonization (e.g., *fim*, *ebp*, *acm*, and *csg* clusters), capsule biosynthesis (*cps* operon, *kpsD/M*), toxin and enzyme production (*astA*, *gelE*, and *sprE*), iron acquisition systems (*ent*, *fep*, *chu*, *shu*, and *iro*), and secretion systems (*esp* family, *bopD*, and *gsp* cluster). Quorum sensing genes (*fsrA-C*) and stress-related factors (icl, *clpP*) were also identified, highlighting broad virulence potential (Table 2). These genes were mainly found in *E. coli* genomes (Figure 4).

### 2.3. Functional Analysis

**Figure 5:**
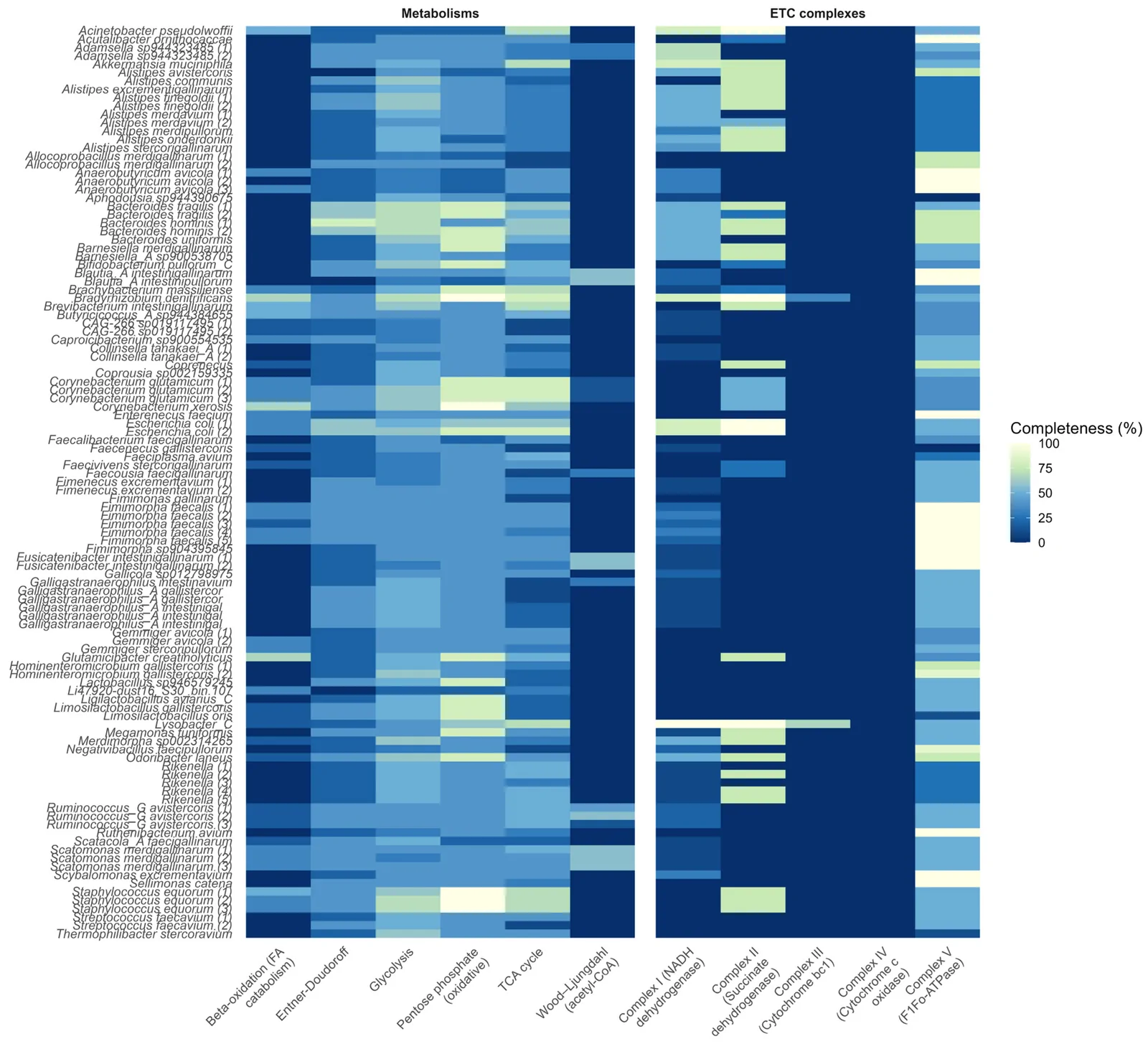
Predicted functional module completeness across MAGs from air. Columns represent metabolic and electron transport chain (ETC) modules, and rows represent individual MAGs labeled according to their GTDB taxonomic assignment. Module completeness was calculated as the percentage of predefined KEGG Orthologs (KOs) detected within each module. Cell color represents completeness from 0 to 100%, with dark blue indicating lower completeness and progressively lighter blue green to pale yellow indicating higher completeness. Modules are separated into metabolism and ETC functional categories.

**Figure 6:**
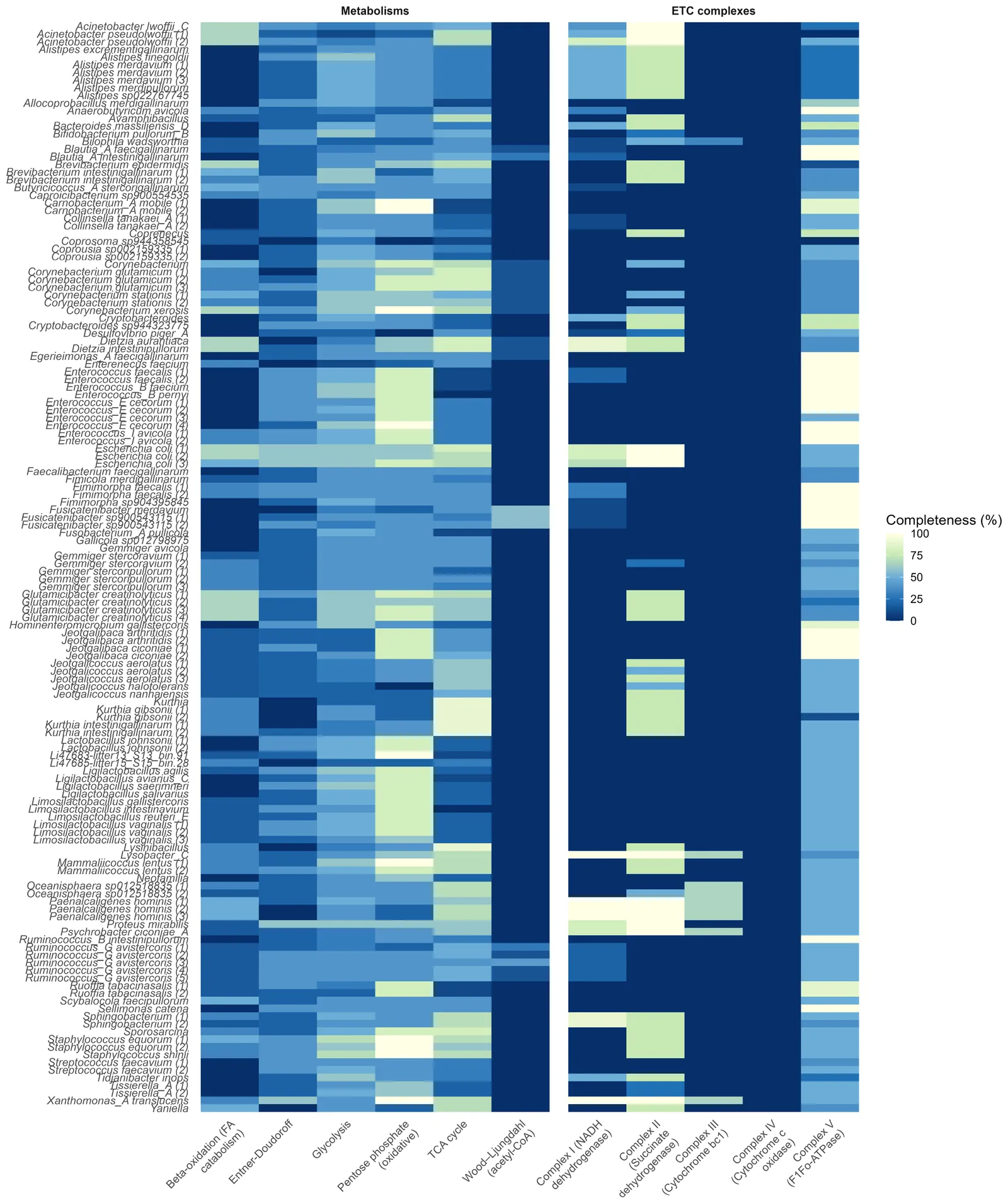
Predicted functional module completeness across MAGs from manure. Columns represent metabolic and electron transport chain (ETC) modules, and rows represent individual MAGs labeled according to their GTDB taxonomic assignment. Module completeness was calculated as the percentage of predefined KEGG Orthologs (KOs) detected within each module. Cell color represents completeness from 0 to 100%, with dark blue indicating lower completeness and progressively lighter blue green to pale yellow indicating higher completeness. Modules are separated into metabolism and ETC functional categories.

**Figure 7:**
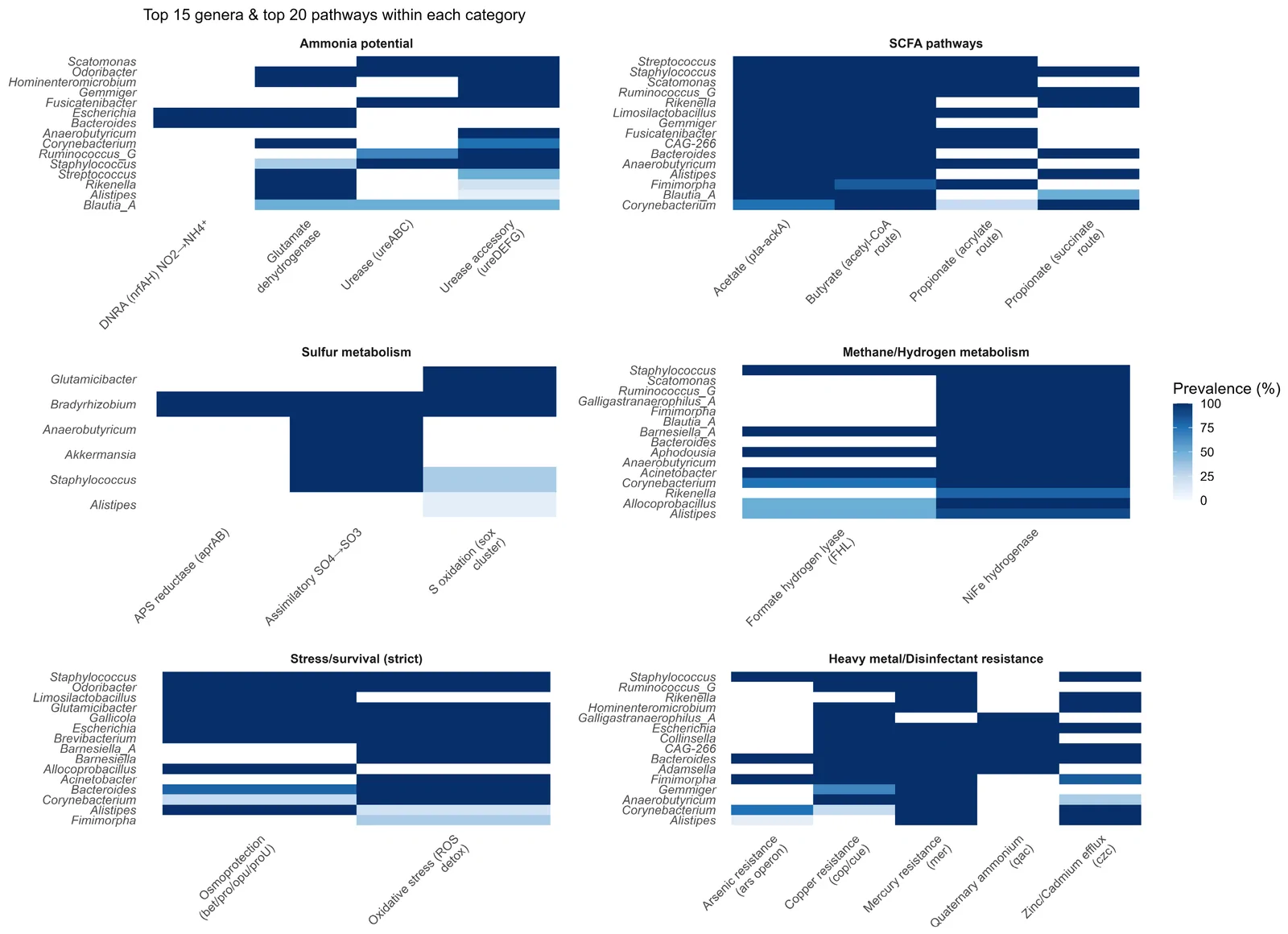
Prevalence of functional pathways across the most represented bacterial genera recovered from MAGs from air. Columns represent functional pathways grouped into their respective functional categories, while rows represent bacterial genera. Cell color indicates pathway prevalence, expressed as the percentage of MAGs within each genus in which the corresponding functional pathway was detected; lighter colors indicate lower prevalence and darker colors indicate higher prevalence.

**Figure 8:**
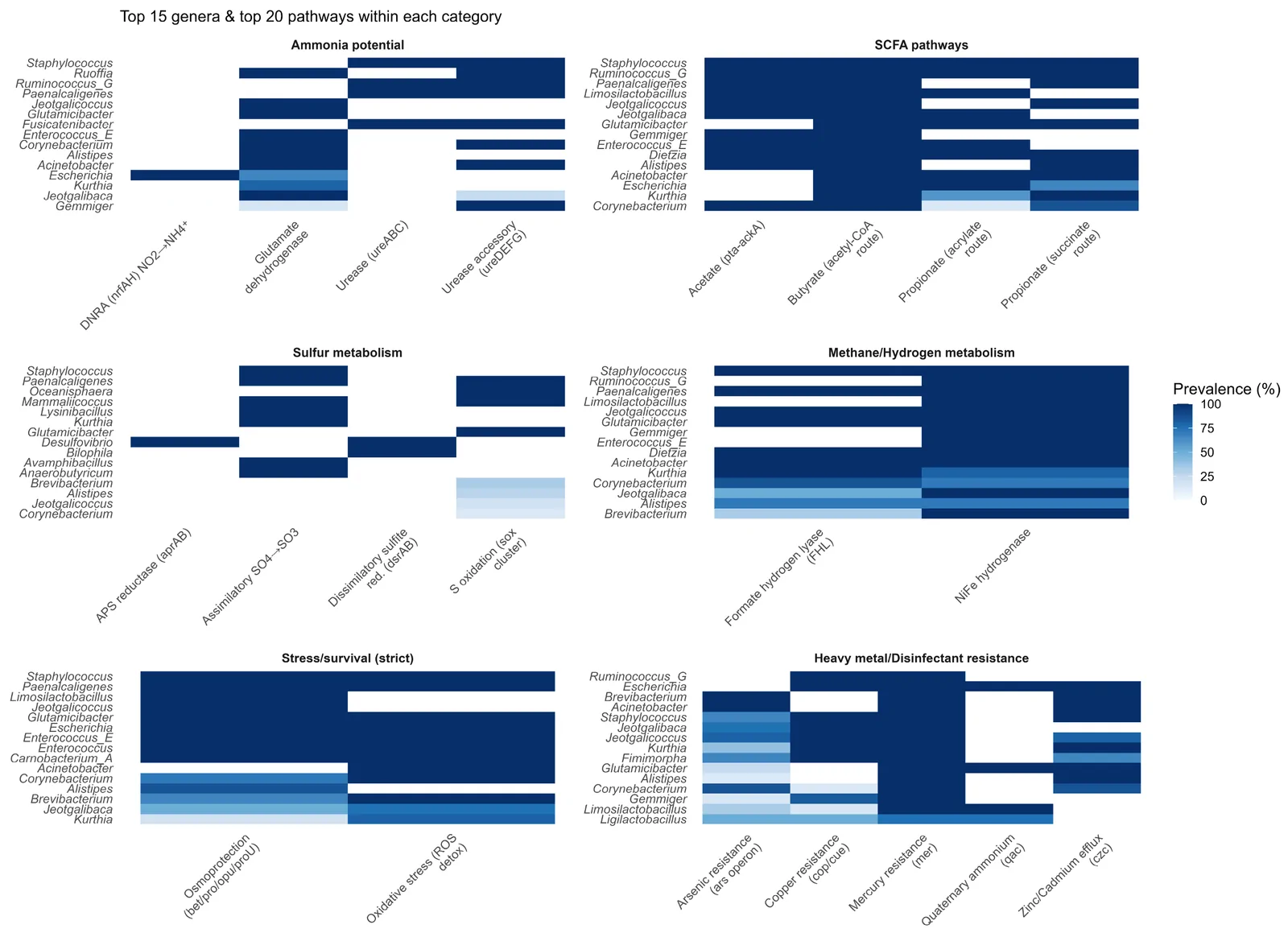
Prevalence of functional pathways across the most represented bacterial genera recovered from MAGs from manure. Columns represent functional pathways grouped into their respective functional categories, while rows represent bacterial genera. Cell color indicates pathway prevalence, expressed as the percentage of MAGs within each genus in which the corresponding functional pathway was detected; lighter colors indicate lower prevalence and darker colors indicate higher prevalence.

High-quality MAGs were also functionally analyzed to study various key aspects of microbial communities for metabolism and ATC complex-related functions from air (Figure 5) and litter (Figure 6). Urease gene (*ureABC*)-related genes were mainly found from both air (Figure 7) and litter (Figure 8) belongingto *Staphylococcus*, *Ruminococcus* and *Corynebacterium* MAGs, while glutamate dehydrogenase-related genes were mainly found in *Alistipes* and *Corynebacterium*. DNRA genes (*nrfAH*) were mainly found in *Escherichia*. All these genes have a role in ammonia production. Meanwhile assimilatory sulfate reduction (*sat*, *cysD/N*) genes responsible for sulfur metabolism and hydrogenase (*NiFe*-type)-related genes responsible for hydrogen cycling were detected from both air and litter from *Staphylococcus.* Butyrate synthesis genes (*acetyl-CoA* route), propionate (succinate/acrylate routes) and acetate (*pta*-*ackA*)-related genes were found in *Staphylococcus* and *Ruminococcus.* Oxidative stress defense systems (*sodA/B*, *katG*, and *ahpCF*) and osmoprotectant uptake systems (*opuA/B/C*, *proU*) were found in many taxa and particularly from all *Staphylococcus* and *Escherichia* genera. Zinc/cadmium efflux (*czc* operons), mercury (*mer*) and Arsenic (*ars*) were distributed across many taxa and found in all *Staphylococcus* genera.

### 2.4. Mobilome Detection

**Figure 9:**
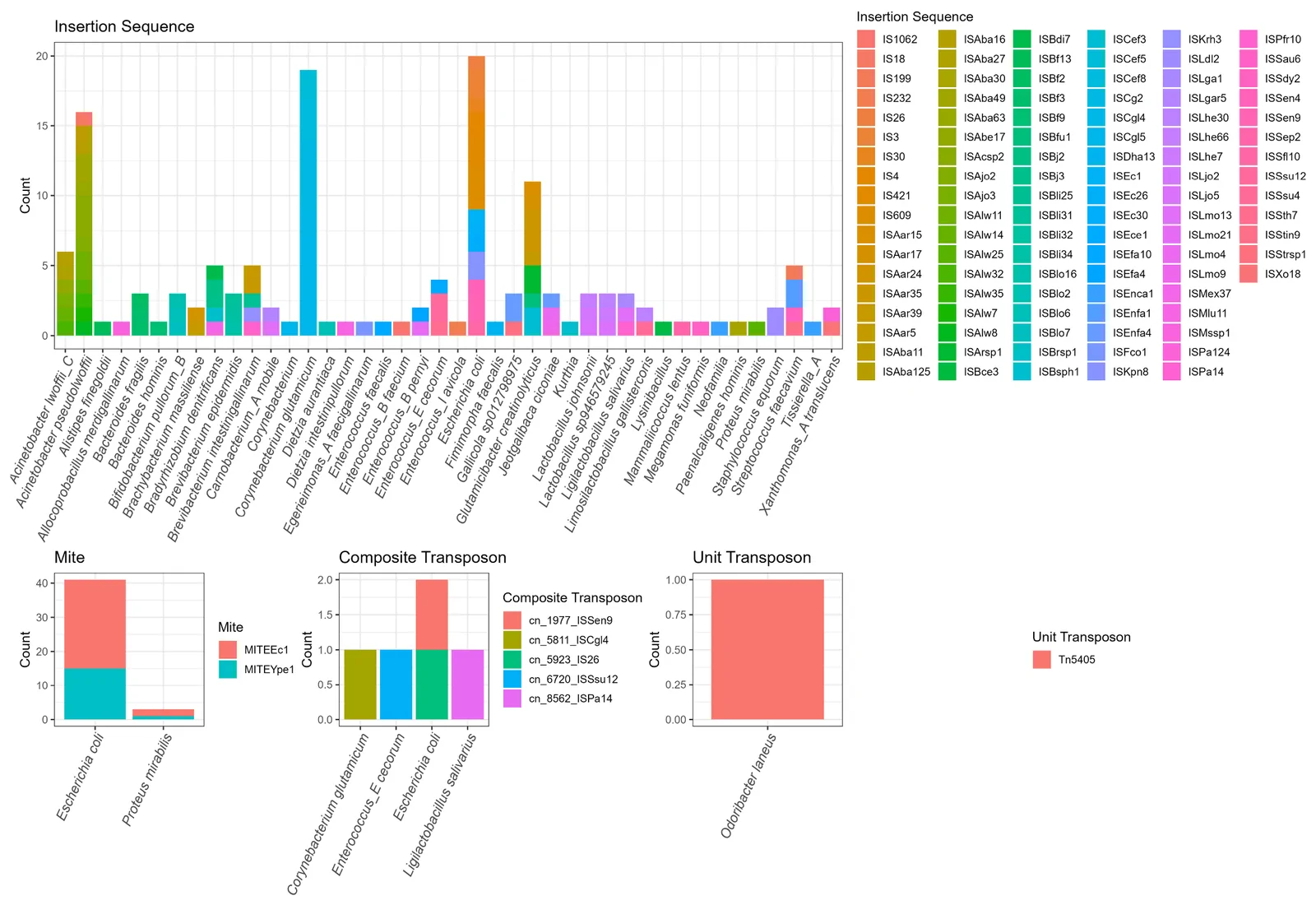
Distribution of mobile genetic elements (MGEs) across MAGs grouped by GTDB taxonomic assignment. The four panels show insertion sequences, miniature inverted-repeat transposable elements (MITEs), composite transposons, and unit transposons. Each stacked bar represents a bacterial taxonomic label, while different colors indicate individual MGE annotations identified using MobileElementFinder. Bar height represents the number of detected MGE occurrences associated with MAGs assigned to each taxonomic group.

**Figure 10:**
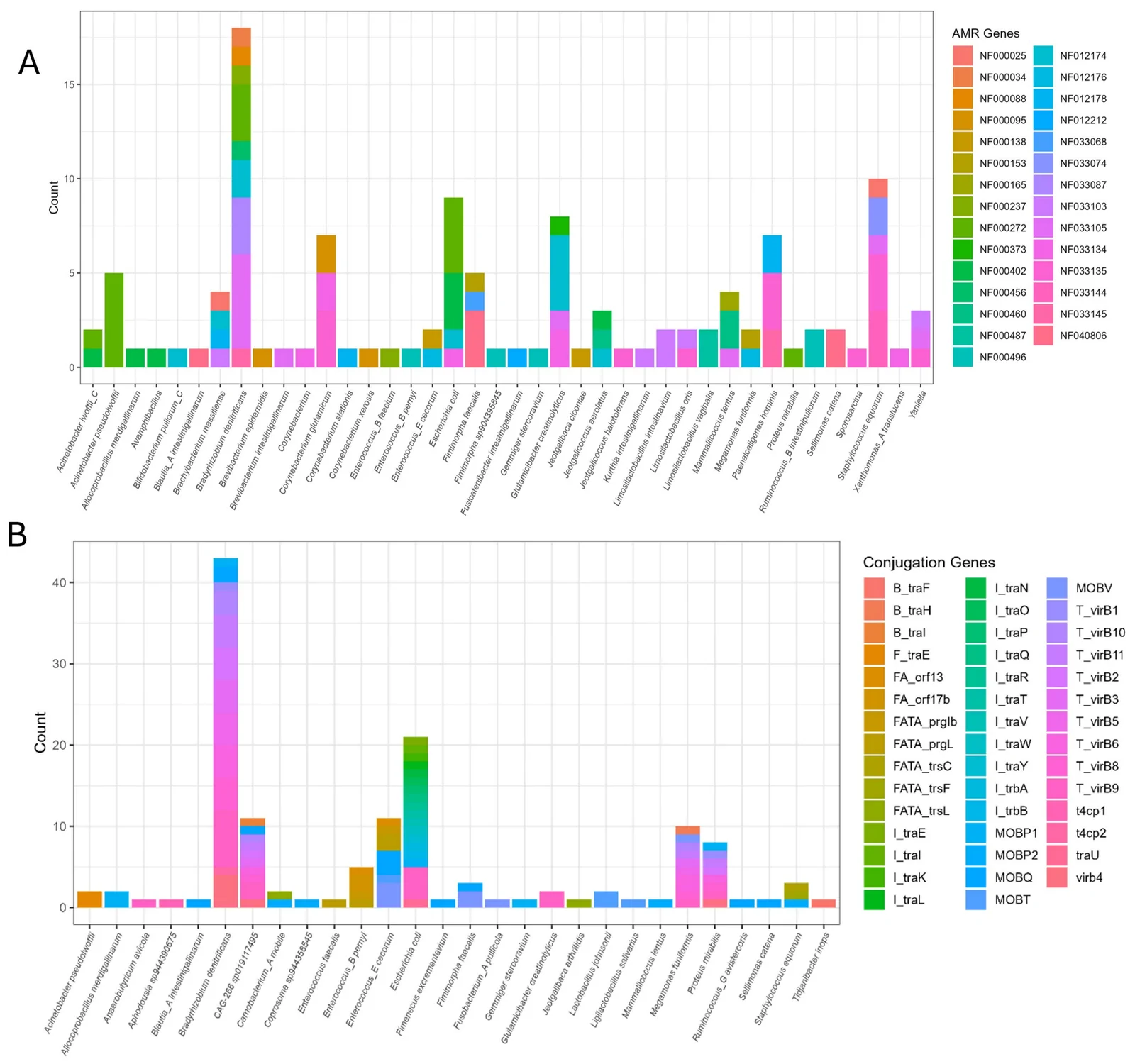
Distribution of predicted plasmid-associated AMR genes across MAGs grouped by GTDB taxonomic assignment. Different colors represent individual AMR gene annotations, and stacked bar height indicates the number of occurrences of these annotations among predicted plasmid sequences associated with each taxonomic group (**A**). Distribution of predicted plasmid-associated conjugation genes across MAGs grouped by GTDB taxonomic assignment. Different colors represent individual conjugation-related gene annotations, and stacked bar height indicates the number of occurrences of these annotations among predicted sequences associated with each taxonomic group (**B**).

Across all MAGs, various MGEs were detected, including plasmids, insertion sequences, composite transposons, unit transposons, transposable elements, and prophages. The detection of these elements differed among bacterial MAGs. Most of the IS were mainly observed in *E. coli*, *Corynebacterium glutamicum*, and *Acinetobacter pseudolwoffii*, while composite transposons were mainly observed in *E. coli*, *Corynebacterium glutamicum*, *E. cecorum*, and *Ligilactobacillus salivarius*, mites were mostly observed in *E. coli* and *Proteus mirabilis*, and unit transposons were mostly observed in *Odoribacter laneus* MAGs. Various types of MGEs are presented according to the respective genomes in Figure 9. Plasmids possessing ARGs (Figure 10A) and conjugate genes (Figure 10B) were mainly present in *Bradyrhizobium denitrificans* and *E. coli* MAGs.

## 3. Discussion

The present study builds upon our previously published metagenomic characterization of poultry barn manure and airborne microbiomes and resistomes [18,19]. Those studies primarily characterized microbial community composition, taxonomic profiles, antimicrobial resistance profiles, and the metagenome-level functional potential of the sequencing datasets. In contrast, the present study provides a genome-resolved analysis of these datasets through assembly, metagenomic binning, MAG quality assessment, genome-level taxonomic classification, and the characterization of antimicrobial resistance, virulence-associated and mobile genetic elements within individual MAGs. This genome-resolved framework enabled the integration of taxonomic, functional, resistance and mobilome-associated features at the level of individual reconstructed genomes, providing information that could not be resolved from community-level metagenomic profiling alone. Bacteria from specific taxa can carry unique and common ARGs and the knowledge about the potential hosts of ARGs subtypes can help us identify the key taxa to focus on when performing risk assessments and for our basic understanding of the ecology and transmission potential of these microbial communities [8,20]. MAGs from shotgun metagenomic sequencing discovered thousands of unique and novel microbial communities carrying subtypes of ARGs and virulence genes, which were not culturable [21].

### 3.1. Network Analysis

It was observed that all *van* gene clusters were only found in litter samples belonging to the *Enterococcus* genus. *Enterococcus* species are normal inhabitants of the gastrointestinal tract of chickens, which can contaminate the food chain and environment [22]. A recent meta-analysis showed a 16% pooled prevalence of vancomycin-resistant *Enterococcus* from chickens throughout the globe [23].

*Lactobacillus* also carried the *str* gene from litter, while *ermB* gene subtypes were found in air. *Lactobacilli* are mainly found in the gut of chickens, and some species are also known to have probiotics potential [24]. The observation of ARGs in commensal organisms is a cause of concern because these are found in large quantities and can spread to the outside environment. *Alistipes* from air samples carried *mef* and *tetQ* gene subtypes. *Alistipes* are also among the main dominant taxa in chicken gut and housing environments [25]. Thay also do have potential to act as probiotics and having resistant against *Alistipes* is a cause of concern. *Lactobacillus* can also carry ARGs, especially for tetracyclines and macrolides, and can act as a reservoir and also play a role in the transfer of ARGs to the food chain [26]. *Staphylococcus* and *Enterococcus* had the most ARGs in the current study.

The genera belonging to *Staphylococcus*, *Enterococcus*, *Mammaliicoccus*, and *Acinetobacter* were the major taxa discovered from litter, while *Staphylococcus*, *Bacteroides*, *Alistipes*, *Streptococcus* and *Rombotsia* were the major taxa originating from air harboring ARG subtypes. *Staphylococcus* was the main genus both from air and manure which carried various types of ARGs, and both *mphC* and *msrA* gene subtypes were found in both sampling sources. Together, these genes can cause resistance against erythromycin. Similar types of finding were also reported from the whole-genome sequencing of NASS from bioaerosols [27]. Most of the genes belong to tetracycline, aminoglycosides and lincosamides from study samples. A study from China and Europe also found that tetracycline, aminoglycoside, and macrolide resistance were predominant and host tracking shows that *Enterococcus* and *Staphylococcus* were among the major taxa carrying subtypes of ARGs [26].

Both archaeal MAGs belonged to *Methanobrevibacter woesei*. *M. woesei* is a symbiont in layer chicken caeca which has a role in gut fermentation, potentially host metabolic regulation, and methane production [28]. Out of 249 high-quality MAGs, 227 were assigned to their respective species levels using the GTDB-tk database, and interestingly 21 MAGs were not assigned to any species and one was not even assigned at the genus level. Various MAGs of *E. coli* carried virulence genes, and avian pathogenic *E. coli* is one of the challenges faced by layer chickens which can cause avian colibacillosis and cause substantial economic losses in layer chickens [29]. Two *E. faecalis* MAGs were also recovered carrying virulence genes, and previously they have been isolated from pullets exhibiting poor uniformity, impaired growth and sporadic lameness from Canada [30]. The detection of diverse virulence genes in poultry barn-associated MAGs reinforces that barn microbiota may harbor traits relevant to colonization, persistence, and potential pathogenicity. For example, *fim* (fimbrial adherence) and capsule biosynthesis genes (*kpsMT*, *cps*) are known in poultry *E. coli* isolates and contribute to host interaction and environmental survival [31,32]. Similarly, *gelE* and *esp*, frequently observed in *E. faecalis* from poultry and shared with human isolates, highlight the capacity for biofilm formation and immune evasion [33,34]. Iron acquisition systems such as *iroN* and *chuA* are also common among avian pathogenic strains, reflecting adaptation to nutrient-limited conditions and contributing to virulence [32,35]. The potential presence of these virulence genes can be a significant threat to the poultry industry, especially when stress conditions are encountered and can be a one health concern. The co-occurrence of ARGs and virulence genes in the same MAGs is important to highlight as they might be potential risk pathogens of one health significance [36]. Though detection alone does not confirm pathogenicity, their presence in potential APCE strains and *Enterococcus* species, which are common in poultry housing environments, needs attention and continuous monitoring.

### 3.2. Functional Analysis

The functional profiles of MAGs across various taxa highlight the potential of various pathways for the production of ammonia, survival, resistance and fermentation, which can have implications for both poultry and humans and fall under the one health umbrella. Ammonia is produced in poultry houses and can have severe implications for poultry and humans during their roles in animal welfare and respiratory distress. The presence of genes encoding for glutamate dehydrogenase across many taxa, including *Enterococcus*, *Alistipes*, *Corynebacterium*, and *Acinetobacter* from litter, indicates the potential for intracellular nitrogen recycling and amino acid catabolism for ammonia release. It highlights the broad metabolic capabilities of microbiota for nitrogen turnover in poultry environments [37,38]. Meanwhile genes related to dissimilatory sulfate reduction found in *Bilophila* and *Desulfovibrio* indicated the potential of microbiota for hydrogen sulfide (H_2_S) production [39]. H_2_S is also a toxic gas with recognized impacts on chicken respiratory health and worker safety [40]. Various management-related practices are being adopted to overcome the production of gases in chicken housing facilities; among them, litter removal and ambient ventilation plays crucial roles [41,42]. Other mitigation approaches involve optimizing crude protein levels and high-fiber foods, balancing sulfur amino acids to limit nitrogen excretion, and supplementation with feed enzymes (e.g., protease, phytase) to enhance digestibility and reduce substrate availability for microbial deamination [43,44,45,46]. Alum and other acidifying litter amendments can also significantly suppress ammonia production from poultry litter [47,48]. More recently, microbiome-informed strategies such as probiotic supplementation and inoculation with nitrifying bacteria are being proposed, though empirical evidence in poultry settings remains limited [49,50].

The presence of quaternary ammonium compounds in commensal and potential opportunistic pathogens including *E. coli* is a cause for concern and suggests that microbes can have the potential to survive even after using these disinfectants. This resilience potential highlights the persistence of opportunistic pathogens and ARGs despite implementing various control strategies. Horizontal gene transfer (HGT) can then transfer these ARGs to other microbial communities by various mechanisms including transformation, conjugation, and transduction [51]. Genes for *czc*, *mer*, and *ars* were also found in *Kurthia* and *Staphylococcus*. Because these systems are often reported to be co-located with AMR determinants, their presence suggests possible co-selection pressure. This finding stresses the need to carefully evaluate metal supplementation and disinfectant rotation practices. The widespread detection of genes with potential for oxidative stress defenses, Osmo protectants, and biofilm operons suggests that many barn microbes are well adapted to survive routine cleaning, aeration, and fluctuations in humidity and temperature. This resilience may underline the persistence of potential pathogens despite control measures. A high prevalence of butyrate and propionate pathways indicates substantial fermentation activity, particularly in litter-associated MAGs. SCFA production may acidify local environments and influence pathogen survival. Butyrate has been linked to pathogen exclusion in gut systems; its presence in litter microbiota may similarly influence microbial dynamics.

Microbiome-informed strategies can offer additional opportunities to tackle issues of poultry housing environments. Probiotics use is gaining attention as an alternative to antibiotics, but apart from that, they can also play a role in suppressing the dominance of taxa responsible for the production of ammonia and other gases [52]. Moreover, nitrifying bacteria inoculation can promote aerobic conversion of ammonia to nitrate and then ultimately lower its emission [49].

### 3.3. Mobilome Analysis

Various mobilome-related genes were observed across multiple MAGs; some of them were conserved to specific taxa while others appeared to be genome-specific, which demonstrates the potential of high genomic plasticity among various bacterial species inhabiting poultry housing environments [11]. They highlight the adaptive potential of these commensal and opportunistic bacterial species. *E. coli* genomes were the main hotspot for all types of mobilomes, virulence and ARGs, highlighting their potential role in HGT events, which has previously been reported to be harbored from chicken [53]. The co-existence of ARGs and MGEs across various commensal bacteria also provides evidence for the potential persistence and spread of AMR in poultry housing environments. Our previous studies also reported that commensal species harbor genes for quaternary ammonium compound-based disinfectants along with ARGs, which further supports the persistence of resistance in the ecosystem [18,27]. The study findings highlight that various bacterial species harbor a diverse range of mobilizable resistome, which can serve as reservoirs for the horizontal gene transfer of these elements within the barn bacteriome and potentially beyond via airborne dissemination and the application of litter in agriculture fields.

The current study has some limitations including a limited sample size, which was restricted to 15 barns, as the sampling was conducted during highly pathogenic avian influenza (HPAI) outbreaks. The relatively limited number of sampled barns and the absence of a priori power calculation restrict the statistical generalizability of the findings. Therefore, the present results should be considered to be exploratory and descriptive and should not be interpreted as capturing the full biological variation present across poultry production environments. Larger studies encompassing more farms, geographic locations, production systems, and environmental conditions will be necessary to validate and generalize these observations. The analysis of MAGs provides insights into the potential of microbial communities, so care should be taken while interpreting the results. Future studies, focusing on transcriptomics and gene expressions, can better help us understand the real-time role of these microbial communities in poultry production systems.

## 4. Materials and Methods

This study included 15 air and 15 manure samples collected from the same layer chicken barns across Alberta, Canada. Sampling was conducted during a period when access to poultry facilities was constrained by HPAI-related biosecurity restrictions. Consequently, sampling was limited to 15 accessible poultry barns. No a priori statistical power analysis was performed to determine the number of barns. The study was designed as an exploratory and descriptive genome-resolved metagenomic investigation rather than a hypothesis-driven study requiring a predefined statistical sample size. Data regarding ventilation rates, temperatures and relative humidity were not consistently recorded during sample collection and therefore could not be retrospectively included. The detailed sampling methodologies for sample collection, DNA extraction, shotgun metagenomic sequencing and certain bioinformatics analysis is presented in previous publications which included 15 air [18] and 15 manure samples [19] processed for shotgun metagenomic sequencing. Air samples were collected using an XMX-CV air sampler (Dycor Technologies, Edmonton, AB, Canada) operated at a primary flow rate of 530 L min^−1^ for 25 min.

### 4.1. Binning, Taxonomic Classification and Network Analysis of ARGs with Taxonomy

The metagenomic samples were assembled using MEGAHIT v1.2.9 (Shenzhen Institutes of Advanced Technology, Chinese Academy of Sciences, Shenzhen, China) followed by binning of the contig files using METABAT2 v2.15 (Lawrence Berkeley National Laboratory, Berkeley, CA, USA) [54]. After the initial binning, the bins were not subjected to automated refinement, manual refinement, or manual curation. The quality of the MAGs was then checked with CheckM v1.2.3 (University of Queensland, Brisbane, Australia), and MAGs with medium (50% completeness and less than 10% contamination) and high quality (90% completeness and less than 5% contamination) were used for further analysis [55]. Mean coverage was estimated by mapping quality-filtered reads to the assembled contigs. Following MAG quality filtering based on completeness and contamination, the retained MAGs had a minimum mean coverage of 13×. This value represents the lowest observed coverage among the retained MAGs rather than a predefined coverage threshold. Medium- and high-quality taxonomic bins were assigned to the MAGs using the GTDB-Tk v2.3.0 (Australian Centre for Ecogenomics, The University of Queensland, Brisbane, QLD, Australia) database [56]. The resistome analysis was conducted using the AMRFinderPlus v3.11.28 (National Center for Biotechnology Information, National Library of Medicine, National Institutes of Health, Bethesda, MD, USA) database on medium- and high-quality bins [57]. Bins containing the taxa and ARGs were then used to carry out the co-occurrence analysis of gene–host interaction networks. A Python v3.13 (Python Software Foundation, Wilmington, DE, USA) script was used to associate the bin taxonomy and ARGs to carry out the network analysis. Various packages in R were used to visualize the network analysis. Virulence genes were then screened using ABRicate v1.0.1 (The University of Melbourne, Melbourne, VIC, Australia) with the VFDB (Institute of Pathogen Biology, Chinese Academy of Medical Sciences and Peking Union Medical College, Beijing, China) database for high-quality genomes [58]. ARGs were reported at the genus level because they were detected across a broader range of MAGs, and genus-level aggregation provided a consistent and sufficiently supported representation of their distribution while avoiding overinterpretation of species-level assignments in draft MAGs. In contrast, virulence-associated genes were detected in a more limited number of MAGs, for which species-level assignments were available for the high-quality MAGs used in this analysis.

### 4.2. Functional Analysis

Genomes were annotated with Prokka (Victorian Bioinformatics Consortium, Monash University, Clayton, VIC, Australia) [59] and then the functional annotations for predicted proteins were done with eggNOG-mapper eggNOG-mapper v2.1.9 (Centro de Biotecnología y Genómica de Plantas, Universidad Politécnica de Madrid–INIA, Madrid, Spain) with default settings [60] to assign functional categories, orthologs, and KEGG (Kyoto Encyclopedia of Genes and Genomes; Kanehisa Laboratories, Kyoto, Japan)/COG (Clusters of Orthologous Genes; National Center for Biotechnology Information, National Library of Medicine, National Institutes of Health, Bethesda, MD, USA) annotations [61,62]. The annotations were then parsed to identify various genes involved in key ecological processes mainly relevant to poultry housing environments. The genes for ammonia potential, sulfur metabolism, methane/hydrogen metabolism, SCFA pathways, stress/survival systems, heavy metal and disinfectant resistance were parsed to see their role in poultry housing environments. For each category, presence/absence matrices were then generated at individual MAG levels and visualized as faceted tile heatmaps by R and R studio.

### 4.3. Mobilome Analysis

A combination of tools was used to characterize the mobile genetic elements (MGEs) in MAGs. geNomad v1.11.1 (National Center for Biotechnology Information, National Library of Medicine, National Institutes of Health, Bethesda, MD, USA) with geNomad database v1.9 with default settings was used to identify the plasmids possessing ARGs and conjugate genes and proviruses [63]. The geNomad end-to-end workflow was applied to each high-quality MAG using the default classification and filtering settings. The number of computational threads was adjusted according to the available resources. A default minimum classification score of 0.7 was used, and no additional user-defined filtering thresholds were applied. MobileElementFinder (mge_finder) v1.1.2 (Center for Genomic Epidemiology, Technical University of Denmark, Kongens Lyngby, Denmark) with MGEdb v1.1.1 and BLAST+ v2.17.0+ was run independently from the geNomad analysis on the MAGs nucleotide sequences. MGE classifications were based on the categories returned by the MGEdb database, including insertion sequences, MITEs, composite transposons, and unit transposons. ARGs, conjugation-associated genes, and plasmid sequences were analyzed independently. Co-occurrence of an ARG or conjugation-associated gene and a predicted plasmid within the same MAGs was not interpreted as evidence of physical linkage or plasmid localization. No sequence-level co-localization analysis was performed; therefore plasmid–ARG and plasmid–conjugation-gene associations were not inferred. Visualization was conducted in R v4.2.1 (R Foundation for Statistical Computing, Vienna, Austria) (tidyverse/ggplot2) for the distribution of mobilome across samples.

## 5. Conclusions

MAGs were reconstructed and analyzed from poultry barn air and manure, presenting a genome-resolved view of microbial diversity, functions, resistome, and virulome potential in these environments. Our investigation discovered that microbiota had a variety of ARGs and metabolic pathways linked to the emission of gases and virulence genes, underscoring their complex roles in barn environments. These findings provide a foundation for developing targeted mitigation strategies, including microbiome-informed interventions, litter/manure amendments, and management practices to reduce both AMR dissemination and environmental emissions. Future studies can further extend this approach with functional validation experiments to translate these insights into sustainable poultry production practices.

## Figures and Tables

**Table 1:** Twenty-two high-quality MAG classifications and ANI to the closest reference genomes.

GTDB-Tk-Based Classification	Closest Reference	ANI to Closest Reference
d__Bacteria;p__Actinomycetota;c__Actinomycetes;o__Actinomycetales;f__Micrococcaceae;g__Yaniella;s__	N/A	N/A
d__Bacteria;p__Bacteroidota;c__Bacteroidia;o__Bacteroidales;f__UBA932;g__Cryptobacteroides;s__	N/A	N/A
d__Bacteria;p__Bacteroidota;c__Bacteroidia;o__Sphingobacteriales;f__Sphingobacteriaceae;g__Sphingobacterium;s__	GCA_019115805.1	94.32
d__Bacteria;p__Bacillota;c__Bacilli;o__Bacillales_A;f__Planococcaceae;g__Sporosarcina;s__	N/A	N/A
d__Bacteria;p__Bacillota_A;c__Clostridia;o__Tissierellales;f__Tissierellaceae;g__Tissierella_A;s__	GCF_029266915.1	81.55
d__Bacteria;p__Pseudomonadota;c__Gammaproteobacteria;o__Xanthomonadales;f__Xanthomonadaceae;g__Lysobacter_C;s__	GCF_021390425.1	93.16
d__Bacteria;p__Actinomycetota;c__Actinomycetes;o__Mycobacteriales;f__Mycobacteriaceae;g__Corynebacterium;s__	N/A	N/A
d__Bacteria;p__Bacteroidota;c__Bacteroidia;o__Sphingobacteriales;f__Sphingobacteriaceae;g__Sphingobacterium;s__	GCA_019115805.1	94.13
d__Bacteria;p__Bacillota;c__Bacilli;o__Bacillales_A;f__Planococcaceae;g__Lysinibacillus;s__	N/A	N/A
d__Bacteria;p__Bacillota_A;c__Clostridia;o__Tissierellales;f__Tissierellaceae;g__Tissierella_A;s__	GCF_029266915.1	81.1
d__Bacteria;p__Bacillota;c__Bacilli;o__Bacillales_A;f__Planococcaceae;g__Kurthia;s__	GCF_003143955.1	91.76
d__Bacteria;p__Bacillota;c__Bacilli;o__Bacillales_D;f__Amphibacillaceae;g__Avamphibacillus;s__	N/A	N/A
d__Bacteria;p__Bacillota;c__Bacilli;o__Lactobacillales;f__Carnobacteriaceae;g__;s__	N/A	N/A
d__Bacteria;p__Bacillota_A;c__Clostridia;o__Tissierellales;f__Peptoniphilaceae;g__Neofamilia;s__	N/A	N/A
d__Bacteria;p__Bacteroidota;c__Bacteroidia;o__Bacteroidales;f__UBA932;g__Coprenecus;s__	GCA_944326245.1	90.86
d__Bacteria;p__Bacteroidota;c__Bacteroidia;o__Bacteroidales;f__Rikenellaceae;g__Rikenella;s__	GCA_019113395.1	88.32
d__Bacteria;p__Pseudomonadota;c__Gammaproteobacteria;o__Xanthomonadales;f__Xanthomonadaceae;g__Lysobacter_C;s__	GCF_021390425.1	93.22
d__Bacteria;p__Bacteroidota;c__Bacteroidia;o__Bacteroidales;f__Rikenellaceae;g__Rikenella;s__	GCA_019113395.1	88.38
d__Bacteria;p__Bacteroidota;c__Bacteroidia;o__Bacteroidales;f__Rikenellaceae;g__Rikenella;s__	GCA_019113395.1	88.38
d__Bacteria;p__Bacteroidota;c__Bacteroidia;o__Bacteroidales;f__Rikenellaceae;g__Rikenella;s__	GCA_019113395.1	87.77
d__Bacteria;p__Bacteroidota;c__Bacteroidia;o__Bacteroidales;f__Rikenellaceae;g__Rikenella;s__	GCA_019113395.1	88.26
d__Bacteria;p__Bacteroidota;c__Bacteroidia;o__Bacteroidales;f__UBA932;g__Coprenecus;s__	GCA_944326245.1	90.91

ANI was calculated using GTDB-Tk, and using FastANI during taxonomic classification.

**Table 2:** Main genes and their virulence-related functions.

Function	Gene Symbol	Role
Adhesion and Colonization	*acm*, *efaA*, *fdeC*, *ompA*, *fimA–I*, *ebpA–C*, *csgB–G*, *pilG*, *pilT*, *yag/ecp cluster*	Host attachment, biofilm formation, persistence
Capsule and Immune Evasion	*cpsA–K*, *kpsD*, *kpsM*	Capsule synthesis, complement resistance
Toxins and Enzymes	*astA*, *gelE*, *sprE*, *aslA*	Tissue invasion, toxin activity
Iron Acquisition	*chuU–W*, *shuA–Y*, *fepA–G*, *fes*, *entA–S*, *iroB*	Heme/iron uptake, siderophore biosynthesis
Secretion Systems	*bopD*, *espK–Y*, *nleD*, *gspC–M*	Effector delivery, host manipulation
Quorum Sensing	*fsrA*, *fsrB*, *fsrC*, *fss1*	Regulation of virulence
Stress and Persistence	*icl*, *clpP*	Intracellular survival, stress response

## Data Availability

The sequences of this project have been submitted to NCBI under submission SRA PRJNA1271599 for manure/litter and PRJNA1231486 for air/dust samples. https://github.com/jjovelc/CareemF_AwaisG_CHM (accessed on 20 June 2025) contains the necessary code for the previous 2 publications. The necessary codes for visualization of MAGs and Supplementary Excel sheets are available at https://github.com/AwaisGhaffarvet/Metagenomic-assembled-genomes (accessed on 20 June 2025).

## Supplementary Materials

Excel sheets for supplementary materials are available at https://github.com/AwaisGhaffarvet/Metagenomic-assembled-genomes (accessed on 25 August 2026).
